# Longitudinal monitoring of cardiac dysfunction with MRI in a mouse model of obesity and type 2 diabetes

**DOI:** 10.1186/1532-429X-16-S1-O113

**Published:** 2014-01-16

**Authors:** Inès Abdesselam, Thomas Troalen, Pauline Pepino, Anne Dutour, Frank Kober, Monique Bernard

**Affiliations:** 1 Aix-Marseille University, CRMBM/CEMEREM CNRS 7339, Marseille, France; 2 Aix-Marseille University, NORT, Inserm 1062, Inra 1260, Marseille, France; 3 Endocrinology, Metabolic Disease and Nutrition, CHU Nord, Marseille, France

## Background

Obesity consequences include type 2 diabetes and cardiovascular diseases. Ectopic fat deposition within certain organs such as heart and liver, have recently been shown to be important independent risk factors for the development of these diseases. The purpose of this study was to perform a longitudinal analysis of the changes in cardio-metabolic functions associated with adiposity increase and insulin resistance development.

## Methods

Male C57BL6R mice were subjected to a high fat high sucrose diet (HFHSD, N = 10) or a regular show diet (RD, N = 10) during 4 months in order to induce obesity and insulin resistance. MRI was performed on a Bruker Biospec AVANCE 4.7T system for animal examination. Mice underwent complete exploration every month as follows: 1/determination of body fat content and distribution including ectopic fat using MRS with PRESS sequence and body imaging, 2/cardiac function assessment with 4-cavity and short-axis cine sequences, and 3/cardiac perfusion investigation with arterial spin labeling [1].

## Results

**Figure 1:**
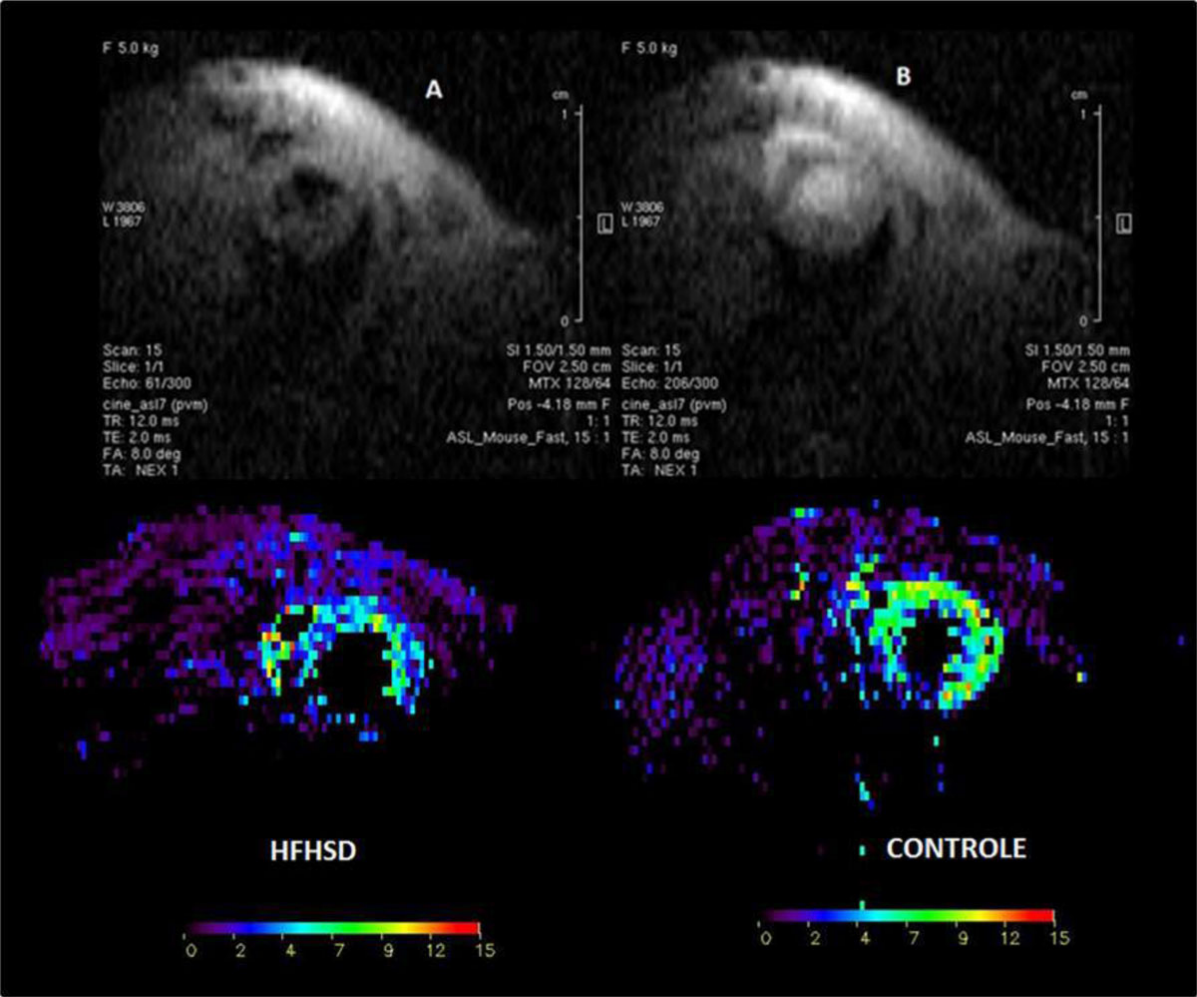


**Figure 2:**
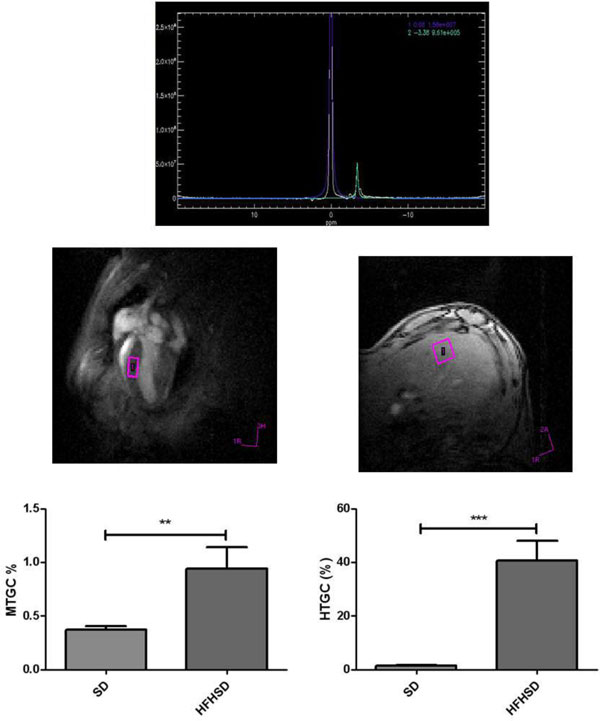


Mice fed with HFHSD became significantly obese compared to mice fed with RD (47.3 ± 0.7 g vs. 32.9 ± 0.1 g). After 4 months of HFHSD, obese mice developed cardiac dysfunctions. Indeed, they presented a significant decrease in end diastolic volume (EDV) compared to control mice (66.9 ± 3.7 mL vs. 85.8 ± 3.0 mL, p = 0.001), stroke volume (SV) (47.4 ± 2.8 mL vs. 58.5 ± 2.8 mL p = 0.01). A significant decrease in cardiac perfusion (7.0 ± 0.7 mL/g/min vs. 9.1 ± 0.6 mL/g/min, p = 0.03) was also observed (Figure 1). These anomalies have been developed subsequently to a significant triglycerides accumulation in heart (1.1 ± 0.20% vs. 0.7 ± 0.08%, p = 0.02) and liver (5.0 ± 1.3x% vs. 1.8 ± 0.09%, p = 0.002) which appears earlier in the 1st month (Figure 2). Furthermore, cardiac perfusion was significantly associated to myocardial (r = -0.5, p = 0.04) and hepatic (r = -0.64, p = 0.008) triglyceride content at 4 months although these two ectopic fats are not correlated together. Interestingly, cardiac perfusion at 4 months was also associated to hepatic triglyceride content at 3 months (r = -0.56, p = 0.03), while no change in cardiac perfusion was still observed. Moreover, increased hepatic triglyceride content was correlated to myocardial function decreases at 4 months: EDV (r = -0.65, p = 0.008), SV (r = -0.64, p = 0.01).

## Conclusions

These preliminary data established the chronological order of anomalies appearance associated to diet-induced obesity and insulin resistance in a 4 month longitudinal assessment. Hepatic triglyceride content seems to be a relevant predictive factor of cardiac anomalies. This experimental protocol might be useful to assess the effect of anti-diabetic drugs on cardiac function.

## Funding

QASAREM.
